# Complete Genome Sequences of Eight *Rhizobium* Symbionts Associated with Common Bean (*Phaseolus vulgaris*)

**DOI:** 10.1128/genomeA.00645-17

**Published:** 2017-07-27

**Authors:** Rosa Isela Santamaría, Patricia Bustos, Olga María Pérez-Carrascal, Fabiola Miranda-Sánchez, Pablo Vinuesa, Irma Martínez-Flores, Soledad Juárez, Luis Lozano, Esperanza Martínez-Romero, Miguel Ángel Cevallos, David Romero, Guillermo Dávila, Ernesto Ormeño-Orrillo, Víctor González

**Affiliations:** Centro de Ciencias Genómicas, Universidad Nacional Autónoma de México, Cuernavaca, Morelos, Mexico

## Abstract

We present here the high-quality complete genome sequences of eight strains of *Rhizobium*-nodulating *Phaseolus vulgaris*. Comparative analyses showed that some of them belonged to different genomic and evolutionary lineages with common symbiotic properties. Two novel symbiotic plasmids (pSyms) with *P. vulgaris* specificity are reported here.

## GENOME ANNOUNCEMENT

There is ample diversity of *Rhizobium* species able to form nodules and fix nitrogen in symbiosis with common bean (1). Although they are commonly referred to as *Rhizobium etli* and *Rhizobium phaseoli*, they may correspond to distinct genomic lineages (2–4). To identify them, we obtained their complete genome sequences. *Rhizobium* isolates were from common bean nodules from different places in Mexico and elsewhere.

Genome sequences were generated with genomic DNA purified with the QIAamp DNA minikit (Qiagen). Illumina MiSeq 2 × 250 and 454 (MOgene LC, St. Louis, MO, USA) technologies were used for DNA sequencing of libraries with inserts of about 200 bp, 2 kb, and 3 kb in length. Paired-end reads were assembled using Newbler 2.5.3 (Roche), Velvet 1.1.06 (5), SSPACE-Basic 2.0 (6), and Consed version 23 (7), with deep coverage between 81 and 529×. Assemblies were guided partially with reference to the complete genomes of *Rhizobium etli* CFN42 and *Rhizobium phaseoli* CIAT652 and with NUCMER alignments (8). Additional long PacBio sequence reads were generated to verify the assembly of the Bra5 and IE4771 genomes. Open reading frames (ORFs) were predicted with Glimmer 3 (9); ORF frame corrections and manual annotations were done in Artemis 12.0 (10) and compared with GenBank (11), InterPro (12), and ISfinder (http://www-is.biotoul.fr) databases.

Table 1 summarizes the genomic features and the GenBank accession numbers of each genome. The lengths of the genomes range from 6.4 to 7.3 Mb, with an overall G+C content of 61%. There are between 3 and 5 plasmids per strain, with sizes ranging from 166 to 1,144 kb. The symbiotic plasmids, or pSyms, are 393 to 530 kb in length, with 59% average G+C content.

**TABLE 1:** General features of *Rhizobium* complete genomes

Species	Strain	Origin	Genome length (bp)	No. of chromosomes, no. of plasmids	G+C content (%)	GenBank accession no.	Reference
*R. etli*	NXC12	Huautla, Mexico	6,756,853	1, 5	61	CP020906 to CP020911	14
*R. phaseoli*	Bra5	Brazil	6,665,454	1, 4	61	CP020896 to CP020900	1
*Rhizobium* spp.	IE4803	Puebla, Mexico	6,997,434	1, 4	61	CP007641 to CP007645	15
	Kim5	Idaho, USA	6,817,255	1, 4	61	CP021124 to CP021128	16
	CIAT894	Colombia	6,657,947	1, 5	61	CP020947 to CP020952	1
	TAL182	Hawaii, USA	6,402,377	1, 5	61	CP021024 to CP021029	1
	IE4771	Puebla, Mexico	7,057,405	1, 5	61	CP006986 to CP006991	15
	NXC14	Huautla, Mexico	6,686,318	1, 3	61	CP021030 to CP021033	14

Average nucleotide identity by MUMmer (ANIm) and a phylogenetic tree, inferred from concatenated nucleotide regions shared among these genomes and others reported in GenBank (13), distinguished Bra5 as belonging to *R. phaseoli* and NCX12 as *R. etli*. Strains Kim5, IE4803, and IE4771 had an ANIm of higher than 95% among them but less than 95% with *R. etli* and *R. phaseoli*. CIAT894, TAL182, and NXC14 were unrelated by ANIm and phylogenetically divergent from other *Rhizobium* species. Therefore, they might represent other genomic lineages and species within the *Rhizobium* genus. The pSyms of IE4803, Bra5, Kim5, TAL182, and CIAT894 are quite similar (ANIm, about 97 to 98%), while strains NXC12 and NXC14 have common pSyms (98% ANIm) but are distinct from the IE4771 pSym that is unique in this collection. Extensive genomic comparisons within the *Rhizobium* genus point out the ecological convergence toward nodulation of a common host due to the presence of similar and divergent pSyms.

### Accession number(s).

Table 1 shows the GenBank accession numbers of the eight strains.
